# Validation Study of Existing Gene Expression Signatures for Anti-TNF Treatment in Patients with Rheumatoid Arthritis

**DOI:** 10.1371/journal.pone.0033199

**Published:** 2012-03-21

**Authors:** Erik J. M. Toonen, Christian Gilissen, Barbara Franke, Wietske Kievit, Agnes M. Eijsbouts, Alfons A. den Broeder, Simon V. van Reijmersdal, Joris A. Veltman, Hans Scheffer, Timothy R. D. J. Radstake, Piet L. C. M. van Riel, Pilar Barrera, Marieke J. H. Coenen

**Affiliations:** 1 Department of Medicine, Radboud University Nijmegen Medical Centre, Nijmegen, The Netherlands; 2 Department of Human Genetics, Radboud University Nijmegen Medical Centre, Nijmegen, The Netherlands; 3 Department of Rheumatology, Radboud University Nijmegen Medical Centre, Nijmegen, The Netherlands; 4 Department of Rheumatology of the St Maartenskliniek, Nijmegen, The Netherlands; University of Bergen, Norway

## Abstract

So far, there are no means of identifying rheumatoid arthritis (RA) patients who will fail to respond to tumour necrosis factor blocking agents (anti-TNF), prior to treatment. We set out to validate eight previously reported gene expression signatures predicting therapy outcome. Genome-wide expression profiling using Affymetrix GeneChip Exon 1.0 ST arrays was performed on RNA isolated from whole blood of 42 RA patients starting treatment with infliximab or adalimumab. Clinical response according to EULAR criteria was determined at week 14 of therapy. Genes that have been reported to be associated with anti-TNF treatment were extracted from our dataset. K-means partition clustering was performed to assess the predictive value of the gene-sets. We performed a hypothesis-driven analysis of the dataset using eight existing gene sets predictive of anti-TNF treatment outcome. The set that performed best reached a sensitivity of 71% and a specificity of 61%, for classifying the patients in the current study. We successfully validated one of eight previously reported predictive expression profile. This replicated expression signature is a good starting point for developing a prediction model for anti-TNF treatment outcome that can be used in a daily clinical setting. Our results confirm that gene expression profiling prior to treatment is a useful tool to predict anti-TNF (non) response.

## Introduction

Rheumatoid arthritis (RA) is a chronic inflammatory disease, which predominantly involves synovial joints and affects up to 1% of the world’s population [1]. Tumour necrosis factor (TNF) neutralization is one of the most effective therapeutic strategies in RA. Nonetheless, this approach is not universally effective and approximately 30% of patients treated with TNF blocking agents fail to achieve or maintain clinical improvement [2]. The combination of prolonged high disease activity, high costs and risk for adverse effects in these non-responding patients has driven the search for predictive markers – including genetic markers – that are able to predict treatment outcome. Insight into the genetics of anti-TNF therapy may facilitate the choice for the most suitable therapy for an individual patient regarding efficacy and safety, thus leading to more individualized treatment in daily clinical practice [3].

In recent years, genome-wide gene expression analysis using microarrays has become a key component in unravelling the underlying transcriptional regulation of various complex diseases [4]-[7]. Gene expression profiling studies in patients with RA have not only revealed genes associated with the disease itself but also identified molecularly distinct subgroups of RA patients [8]-[11]. Gene expression microarray technology has also shown to be able to assist in identifying genes which are involved in treatment response or adverse events associated with therapy [12]-[16]. To date, several studies used genome-wide gene expression analysis to identify gene expression signatures predicting the response to anti-TNF treatment in patients with RA [8], [17]–[28]. Lequerré and co-workers investigated peripheral blood mononuclear cell (PBMC) derived RNAs from 13 RA patients treated with infliximab by the use of a custom made microarray covering 10,000 non-redundant human cDNAs. Expression levels prior to treatment initiation of 41 mRNAs were identified that perfectly separated subsequent responders (n = 6) from subsequent non-responders (n = 7) to infliximab. Validation in 20 other patients reduced the set to 20 transcripts which classify anti-TNF responders and non-responders with a sensitivity of 90% and a specificity of 70%. Further reduction of the transcript set to only 8 transcripts changed sensitivity to 80% and specificity to 100% [19]. More recently, Julia *et al*. (44 patients) and Tanino *et al*. (42 patients) reported, using white blood cells, an eight-gene and a ten-gene expression signature predictive for anti-TNF response, respectively [22], [23]. Stühlmuller and coworkers reported gene sets consisting of 82, 11 and 3 genes as predictive for anti-TNF response [28]. One of the genes in these sets (CD11c) could discriminate responders from non-responders with a sensitivity of 100% and a specificity of 91.7%. Koczan and co-workers analyzed RNA extracted from PBMCs three days after treatment initiation of 19 RA patients treated with etanercept. Forty-two differentially expressed genes were examined for their ability to discriminate between anti-TNF responders and non-responders, reaching prediction accuracies of 95% [18].

Similar studies have been performed using arthroscopic biopsies as RNA source. Lindberg *et al.* examining 10 RA patients, revealed 279 genes significantly differentially expressed in responders and non-responders to infliximab [17]. Badot *et al*. analyzed 25 patients an identified an expression signature of 439 genes to be associated with poor response to anti-TNF therapy [24]. A large study including biopsies of 65 patients could not identify an expression profile predictive of treatment outcome [25].

Other studies used expression profiling to get more insight into the mechanisms underlying the action of anti-TNF [20], [21], [27]. They suggest that responders to treatment are characterized by a higher expression of inflammatory genes in synovial tissue [20] and that the increased expression of inflammatory genes in responders normalizes faster than in non-responders [21]. Baarsen and colleagues showed that TNF treatment resulted in downregulation of genes in diverse immune related pathways including inflammation, angiogenesis, B- and T-cell activation [26]. In a second study they suggest that patients not responding to anti-TNF treatment show an increase in expression of type I interferon response genes [27].

Despite these promising results, the genes identified in each study show little overlap. This can partly be caused by the high false positive rate associated with multiple testing in a limited sample, thus necessitating validation in separate cohorts. In this report we used gene expression profiling on whole blood from 42 RA patients treated with the monoclonal anti-TNF antibodies infliximab or adalimumab to validate previously reported gene expression signatures [19], [21]–[23], [28] for their predictive value in our independent cohort of RA patients treated with anti-TNF.

## Results

Table 1 shows patients’ characteristics, mean disease activity (DAS28) at baseline and DAS28 improvement 14 weeks after treatment start. In total 42 RA patients treated with anti-TNF were included in the study. According to the EULAR definition of response [29], 18 patients in our sample responded well to anti-TNF treatment and 24 patients showed no response to the treatment. Twenty-seven patients were treated with infliximab, 15 were treated with adalimumab. No differences in patient characteristics were observed between the responder and non-responder groups except for DAS28 improvement after 14 weeks of treatment. No differences in WBC numbers (lymphocytes, neutrophils, eosinophils, basophils and monocytes) were observed between the responder and non-responder group (data not shown).

**Table 1:** Baseline characteristics, disease activity at baseline and DAS28 improvement for responders and non-responders to anti-TNF treatment.

	Responders	Non-responders	P-value
N (baseline and 14 weeks follow-up)	18 (43%)	24 (57%)	NS
Female gender	16 (89%)	14 (58%)	NS
Age (mean±SD)	58±14.2	57±13.6	NS
RF positivity	13 (72%)	19 (79%)	NS
Adalimumab	4 (27%)	11 (52%)	NS
Infliximab	14 (73%)	13 (48%)	NS
MTX-comedication	18 (100%)	24 (100%)	NS
DAS28 baseline (mean±SD)	5.3±1.0	4.8±1.5	NS
DAS28 decrease after 14 weeks of anti-TNF therapy (mean±SD)	2.0±0.8	0.1±1.0	<0.0001

Results are number (percentage) or mean (SD). Percentages are expressed in relation to the total number of patients for each response group (except for the total number of patients). P-value indicates a significant difference between the two response groups NS: not significant.

**Figure 1:**
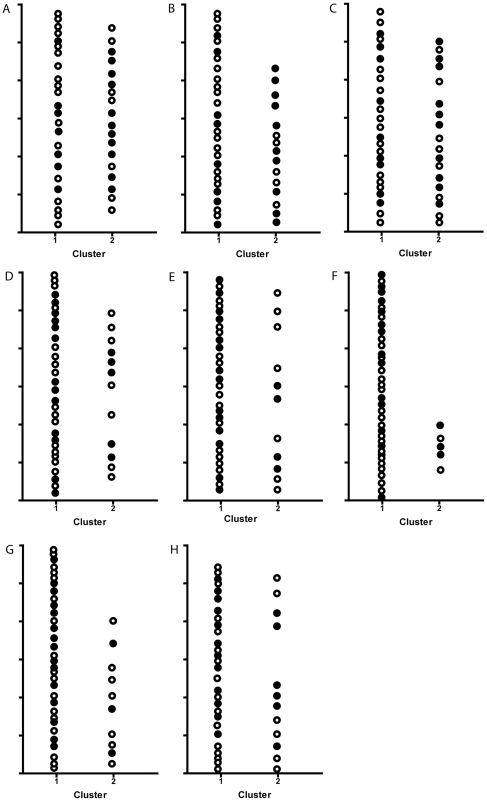
Cluster analysis for the reported transcript sets. K-means cluster analysis based upon the transcript sets reported by (A) Lequerré (20 genes) (B) Stuhlmuller (11 genes) (C) Stuhlmuller (82 genes) (D) Lequerré (8 genes) (E) Sekiguchi (18 genes) (F) Julia (8 genes) (G) Stuhlmuller (3 genes) and (H) Tanino (8 genes). The previously published transcript sets were linked to the expression values of 42 RA patients treated with anti-TNF in our study. The two clusters were identified as the non-responder (1) and responder (2) clusters. Profiles are ranked according the results obtained after clustering, in which profile A showed the best results. • = responder; ○ = non-responder.

Expression profiling on whole blood from these 42 RA patients was performed to generate a whole-genome expression dataset. We used this set to validate data from five previously published studies by extracting the expression levels observed for the genes reported by them from our dataset [19], [21]–[23], [28]. In total eight transcript sets from the studies were linked to the expression values of our 42 RA patients followed by K-mean clustering (Figure 1). After clustering, the sensitivity and specificity for each of these transcripts was calculated (Table 2). The best result was obtained by the 20 genes transcript set of Lequerré *et al*. This set was able to classify our patients as anti-TNF responders and non-responders with a sensitivity of 71% and a specificity of 61% (Figure 1A). Although the other transcript sets also reached reasonable sensitivities (ranging from 92% to 67%), the specificities were low (ranging from 56% to 17%) (Table 2). Next, we performed an exploratory genome-wide analysis of the data and identified 113 genes that, at baseline, were significantly differentially expressed in responders and non-responders to TNF blockade by monoclonal antibodies (Table S1).

**Table 2:** Sensitivity and specificity for each transcript set.

Study	Reference	Sensitivity (%)	Specificity (%)
Lequerré (20 genes)	[19]	71	61
Stuhlmuller (11 genes)	[28]	79	56
Stuhlmuller (82 genes)	[28]	67	56
Lequerré (8 genes)	[19]	71	28
Sekiguchi (18 genes)	[21]	71	28
Julia (8 genes)	[23]	92	17
Stuhlmuller (3 genes)	[28]	71	17
Tanio (8 genes)	[22]	67	33

Eight previously published transcript sets were linked to the expression values of 42 RA patients treated with anti-TNF in this study. After k-means cluster analysis the sensitivity and specificity were calculated

## Discussion

In this study we used genome-wide expression profiling to validate eight previously reported gene expression signatures predicting anti-TNF therapy outcome. To our knowledge, this is the first study in which previously reported expression signatures for anti-TNF response are re-investigated in an independent patient cohort. This analysis was based on whole transcriptome profiling prior to the first anti-TNF administration.

The expression profiles identified in different studies are often not consistent with each other and different gene sets have been reported to distinguish between responders and non-responders [17]-[19], [21]-[28], [30], [31]. One reason for the differences between studies might be the limited sample sizes and the high rate of false positive findings associated with multiple testing. Other reasons for inconsistent results might be differences in tissues used for analysis (synovial biopsies, PBMCs, whole blood), RNA isolation and analyses at different time points, differences in types and doses of anti-TNF medication, differences in response criteria (ACR, EULAR or DAS28 change), differences in techniques (array platforms, q-PCR methods), differences in patient clinical characteristics (disease activity, gender) and differences in patient ethnicity (Caucasian, Asian). Despite the differences observed between the studies it turns out to be possible to obtain a reasonably good classification of anti-TNF responders and non-responders for one of the eight previously described candidate gene expression profiles. One transcript set (20 genes) from the study of Lequerré *et al.* [19] was validated with a sensitivity of 71% and specificity of 61%. This 20 genes profile results in a relatively good sensitivity and specificity in our data set, even in a different type of material (whole blood versus PBMCs). However, we failed to validate seven other previously described transcript sets.

Though these results can be viewed as a first step towards a diagnostic test, a critical remark is in place. No moderate responders were included in this cohort. Before such an expression profiling test can be implemented in the clinic, validation of these expression signatures in a larger cohort, consisting of good, moderate and non-responders, is needed. Also the obtained sensitivity of 71% and specificity of 61% are not high enough for daily clinical practice. However, the sensitivity and specificity of the tests might be further enhanced by including other types of biomarkers, like genetic polymorphisms, and/or clinical characteristics.

The current analysis should be viewed in the light of some strengths and limitations. A relative strength of our study is the sample size. Our study investigated 42 well characterized patients. To our knowledge, it is for the first time that an expression profiling dataset concerning anti-TNF (non-)response is used to validate other, previously reported expression signatures for predicting anti-TNF response. This leads to a more evidence-based and better argued conclusion in favor of expression profiling as a tool for predicting anti-TNF response then the results from one single experiment. A limitation of the study is given by the fact that RA is a very heterogeneous disease. Individual patient characteristics like RF, DAS28, CRP, disease duration, disease onset, age, co-medication, joint erosions, smoking and Health Assessment Questionnaire (HAQ) will always be slightly different between patients, which makes it very difficult to select two homogeneous patient groups. This will most certainly limit the power to detect gene expression differences between anti-TNF responders and non-responders in diverse patient cohorts.

To conclude, this study successfully validated an earlier reported gene expression profile predictive of anti-TNF treatment outcome. Before this set can be used in clinical practice the predictive value should be increased by adding additional predictors of anti-TNF treatment outcome. However the validated gene-expression profile can be viewed as a starting point to construct a prediction model for anti-TNF treatment outcome.

## Materials and Methods

### Ethics statement

The “Commissie Mensgebonden Onderzoek (CMO) Regio Arnhem Nijmegen” of the Radboud University Nijmegen Medical Centre approved the study (CMO number 2004/014). All patients had provided written informed consent prior to participation in the study. All clinical investigation were conducted according to the principles expressed in the Declaration of Helsinki.

### Patients

All patients had RA according to the 1987 revised American College of Rheumatology (ACR) criteria [32] and attended the Departments of Rheumatology of the Radboud University Nijmegen Medical Centre or the St. Maartenskliniek in Nijmegen. The patients selected for the current study all participate in the Dutch Rheumatoid Arthritis Monitoring (DREAM) registry (www.dreamregistry.nl). The latter collects detailed clinical information and treatment outcome of patients who start their first course of a TNF-blocking agent according to the Dutch recommendations (Disease Activity Score 28 (DAS28)>3.2 and previous failure on at least two disease-modifying antirheumatic drugs (DMARDs), one of which has to be methotrexate (MTX)) [33] Response to TNF neutralization was assessed at week 14 according to the EULAR criteria [29]. Consecutive patients enrolled in the DREAM registry between 2004 and 2008 were included in this study.

Only good responders and non-responders at 14 weeks based on the EULAR response criteria were selected for expression analyses. Patients with a moderate response were excluded. This resulted in forty-two patients (18 good responders and 24 non-responders) that were included in the study (Table 1), representing the extremes of a total of 92 patients. Power calculations showed that this sample of 42 patients had a power of 80% to detect a minimal fold change of two with an alpha of 0.0000027. Responders and non-responders were frequency-matched for gender, age, RF-positivity and use of MTX. Blood was sampled prior to treatment start (infliximab or adalimumab).

### Molecular analyses

All molecular analyses were performed in a CCKL (Coördinatie Commissie ter bevordering van de Kwaliteitsbeheersing van het Laboratoriumonderzoek) -accredited laboratory at the Department of Human Genetics at the Radboud University Nijmegen Medical Centre in Nijmegen. RNA was isolated from whole blood within 0.5 hours after venapuncture, using the RNeasy midi kit according to the manufacturer’s protocol (Qiagen Benelux B.V. Venlo, The Netherlands). To remove residual traces of genomic DNA, the RNA was treated with DNase I (Invitrogen, Leek, The Netherlands) while bound to the RNeasy column. Quality and quantity of the purified RNA was controlled using a NanoDrop spectrophotometer (Nanodrop technologies, Montchanin, DE, USA). RNA integrity was investigated by using the 2100 Bioanalyser (Agilent technologies, Philadelphia, PA, USA). RNA was examined for possible degradation using agarose gel electrophoresis.

Gene expression profiling was performed using Affymetrix 1.0 Human Exon ST arrays, representing all known genes (17881) (Affymetrix Inc., Santa Clara, CA, USA) according to the manufacturer’s instructions. The Affymetrix GeneChip Whole Transcript Sense Target Labeling Assay was used to generate amplified and biotinylated sense-strands DNA targets from the entire expressed genome (2.0 µg of total RNA). Arrays were hybridized by rotating them at 60 rpm in the Affymetrix GeneChip hybridization oven at 45°C for 17 hours. After hybridization, the arrays were washed in the Affymetrix GeneChip Fluidics station FS 450. Arrays were scanned using the Affymetrix GeneChip scanner 3000 7G system.

### Data extraction

For quality control, the Affymetrix CEL-files were first imported into Affymetrix Expression Console version 1.1 where control probes were extracted and normalized using the default RMA algorithm. The Area Under the Curve (AUC) of the Receiver Operator Characteristic was calculated using the positive and negative control probes. All arrays had an AUC score above the empirically defined threshold of 0.85 indicating a good separation of the positive controls from the negative controls. Pearson correlation between arrays showed no outliers.

Subsequently, the CEL-files were imported into Partek® (Partek® Genomic Suite software, version 6.4 Copyright © 2008 Partek Inc., St. Louis, MO, USA) where only core probe sets were extracted and normalized using the RMA algorithm with GC background correction. Core transcript summaries were calculated using the mean intensities of the corresponding probe sets, representing the quantitative expression levels of all genes. Expression data is generated according the MIAME guidelines and is available through the GEO (Gene Expression Omnibus) database (http://www.ncbi.nlm.nih.gov/geo), accession number GSE33377. Genes that showed significantly differential expression in responders and non-responders to TNF blockade by monoclonal antibodies are presented in Table S1.

### Validation of previously reported expression signatures

Using the PubMed database (accessed August 2011), we identified a total of fourteen studies that used gene expression profiling to predict treatment outcome [17]-[28], [31], [34]. Nine studies were excluded from validation as they analyzed gene expression levels after treatment initiation [18], [26], [27], [34] or used different starting material (arthroscopic biopsies) [17], [20], [24], [25], [31]. The transcript sets from the other studies [19], [21]-[23], [28] were included for validation in our patient cohort because they matched our experimental set up in the following aspects: 1) all studies present transcript sets that are able to distinguish between responders and non-responders based upon analyses at baseline (before treatment start) and 2) the studies used blood cells as starting material. The published transcript sets were linked to the corresponding quantitative expression values obtained in our analyses. K-means partition clustering was performed using Pearson dissimilarity as a distance measure. The number of partition clusters was set to two (non-responder and responder). The true positive and true negative responses values were calculated. Sensitivity was calculated by the following formula: true positives (true anti-TNF non-responders identified as non-responders)/true positives+false negatives (true non-responders identified as responders). Specificity was calculated by the formula: true negatives (true responders identified as responders)/true negatives+false positives (true responders identified as non-responders).

## Supporting Information

Table S1 Click here for additional data file. DOC
