# Supplementary material for: Validation Study of Existing Gene Expression Signatures for Anti-TNF Treatment in Patients with Rheumatoid Arthritis
Source: PLoS One. 2012 Mar 21;7(3):e33199. doi: 10.1371/journal.pone.0033199 (PMC3310059; doi:10.1371/journal.pone.0033199)
Supplement: Table S1 [file pone.0033199.s001.doc]

**Table S**1

| **Gene symbol** | **Gene name** | **Chromosomal location** | **Biological process** | **P-value** | **Fold change** |
| --- | --- | --- | --- | --- | --- |
| *AY358807* |  | 6p25.1 | Unknown | 0,0000079314 | -1,1863420234 |
| *HIRIP3* | HIRA interacting protein 3 | 16p11.2 | Chromatin assembly or disassembly | 0,0000590445 | -1,0992046406 |
| *TPM1* | Tropomyosin 1 (alpha) | 15q22.1 | Cell motility | 0,0004030710 | 1,3065841103 |
| *TUSC4* | Tumor suppressor candidate 4 | 3p21.3 | Cell cycle | 0,0004597140 | -1,1389938838 |
| *CLIC3* | Chloride intracellular channel 3 | 9q34.3 | Ion transport | 0,0004779760 | -1,3321347284 |
| *PTGS2* | Prostaglandin-endoperoxide synthase 2 | 1q25.2-q25.3 | Fatty acid biosynthetic process, prostaglandin biosynthesis, inlammation | 0,0005020940 | 1,9983509899 |
| *G0S2* | G0/G1switch 2 | 1q32.2-q41 | Regulation of progression through cell cycle | 0,0006410960 | 2,4122843899 |
| *PIGV* | Phosphatidylinositol glycan anchor biosynthesis, class V | 1p36.11 | GPI anchor biosynthetic process | 0,0006426890 | -1,1346049003 |
| *MOP-1* | MOP-1 | 4q21.22 | Unknown | 0,0006548130 | 2,9017435044 |
| *ZBTB6* | Zinc finger and BTB domain containing 6 | 9q33.2 | Transcription | 0,0008614900 | -1,1988656556 |
| *RANBP17* | RAN binding protein 17 | 5q34 | Protein import into nucleus, docking | 0,0010152600 | 1,0633820361 |
| *PCGF5* | Polycomb group ring finger 5 | 10q23.32 | Transcription | 0,0011237900 | 1,3278111760 |
| *SESTD1* | SEC14 and spectrin domains 1 | 2q31.2 | Unknown | 0,0011555700 | 1,3007556736 |
| *GPD2* | Glycerol-3-phosphate dehydrogenase 2 (mitochondrial) | 2q24.1 | Glucose catabolic process | 0,0011869500 | 1,3649896387 |
| *FLJ30672* | Hypothetical protein FLJ30672 | Xq26.3 | Unknown | 0,0012008000 | -1,0778825609 |
| *HERPUD2* | HERPUD family member 2 | 7p14.2 | Protein modification process | 0,0012987700 | 1,1074027426 |
| *DND1* | Dead end homolog 1 (zebrafish) | 5q31.3 | Multicellular organismal development | 0,0014789900 | -1,4293507654 |
| *SH2D2A* | SH2 domain protein 2A | 1q21 | Angiogenesis, cell differenation, signal transduction | 0,0014843300 | -1,2569001112 |
| *EIF4E2* | Eukaryotic translation initiation factor 4E family member | 2q37.1 | Translational initiation | 0,0015043000 | -1,1357222065 |
| *GTPBP2* | GTP binding protein 2 | 6p21-p12 | Small GTPase mediated signal transduction | 0,0017672300 | 1,2122355023 |

**Table S**1. (continued)

| **Gene symbol** | **Gene name** | **Chromosomal location** | **Biological process** | **P-value** | **Fold change** |
| --- | --- | --- | --- | --- | --- |
| *GPR175* | G protein-coupled receptor 175 | 3q21.2 | Lipid metabolic process | 0,0018316000 | -1,1175504802 |
| *GRAMD1B* | GRAM domain containing 1B | 11q24.1 | Unknown | 0,0018378800 | 1,2787462419 |
| *PPP1R15A* | Protein phosphatase 1, regulatory (inhibitor) subunit 1 | 19q13.2 | Apoptosis, cell cycle arrest, regulation of transcription | 0,0018827800 | 1,3286397662 |
| *PMAIP1* | Phorbol-12-myristate-13-acetate-induced protein 1 | 18q2 | Cytochrome c from mitochondria | 0,0019496400 | 1,6168882820 |
| *RAPGEF1* | Rap guanine nucleotide exchange factor (GEF) 1 | 9q34.3 | Signal transduction, apoptosis | 0,0020499000 | 1,4063054673 |
| *AXUD1* | AXIN1 up-regulated 1 | 3p22 | Apoptosis, regulation of transcription | 0,0021000800 | 1,4273013826 |
| *TMOD2* | Tropomodulin 2 (neuronal) | 15q21.1-q21.2 | Nervous system development | 0,0022829900 | 1,2968395547 |
| *EGR2* | Early growth response 2 | 10q21.1 | Regulation of transcription, apoptosis | 0,0023460600 | 1,5217907518 |
| *DUSP1* | Dual specificity phosphatase 1 | 5q34 | Protein amino acid dephosphorylation | 0,0024407000 | 1,2796949982 |
| *C7orf41* | Chromosome 7 open reading frame 41 | 7p15.1 | Unknown | 0,0025218400 | 1,3379461025 |
| *EGR3* | Early growth response 3 | 8p23-p21 | Transcription, apoptosis | 0,0025907300 | 1,6454589097 |
| *SQSTM1* | Sequestosome 1 | 5q35 | Ubiquitin-dependent protein catabolic process, apoptosis | 0,0026913800 | 1,1062979600 |
| *RAMP3* | Receptor (G protein-coupled) activity modifying protein 3 | 7p13-p12 | Intracellular protein transport, immune response | 0,0028468000 | -1,1917817001 |
| *PDE3A* | Phosphodiesterase 3A, cGMP-inhibited | 12p12 | Lipid metabolic process | 0,0029371300 | 1,4429386885 |
| *VEPH1* | Ventricular zone expressed PH domain homolog 1 (zebrafish) | 3q24-q25 | Unknown | 0,0029713000 | 1,2261623401 |
| *GBP7* | Guanylate binding protein 7 | 1p22.2 | Immune response | 0,0029735800 | 1,2414791221 |
| *PSTPIP2* | Proline-serine-threonine phosphatase interacting protein | 18q12 | Unknown | 0,0030226900 | 1,3439132363 |
| *C7orf46* | Chromosome 7 open reading frame 46 | 7p15.3 | Unknown | 0,0031560700 | -1,0750993608 |

**Table S1. (continued)**

| **Gene symbol** | **Gene name** | **Chromosomal location** | **Biological process** | **P-value** | **Fold change** |
| --- | --- | --- | --- | --- | --- |
| *ZNF2* | Zinc finger protein 2 | 2q11.2 | Transcription | 0,0033255200 | -1,1092541879 |
| *MED12L* | Mediator complex subunit 12-like | 3q25.1 | Transcription, proliferation | 0,0034171100 | 1,1372346801 |
| *OSM* | Oncostatin M | 22q12.2 | Regulation of cell growth, immune response | 0,0034455200 | -1,8719176905 |
| *TMEM186* | Transmembrane protein 186 | 16p13 | Unknown | 0,0034506000 | -1,2857587752 |
| *PKHD1L1* | Polycystic kidney and hepatic disease 1 | 8q23.1-q23.2 | Unknown | 0,0036954900 | 1,2912783287 |
| *OR6C74* | Olfactory receptor, family 6, subfamily C, member 74 | 12q13.13 | Signal transduction | 0,0039173400 | -1,1065126925 |
| *ATPBD1B* | ATP binding domain 1 family, member B | 1p36.11 | Unknown | 0,0041743900 | -1,0981613185 |
| *BAT1* | HLA-B associated transcript 1 | 6p21.3 | Nuclear mRNA splicing, via spliceosome | 0,0041884300 | -1,0572085517 |
| *AY358772* |  | 3q13.2 | Unknown | 0,0042496200 | -1,1987327045 |
| *C15orf40* | Chromosome 15 open reading frame 40 | 15q25.2 | Unknown | 0,0044121300 | -1,1210417436 |
| *CMIP* | C-Maf-inducing protein | 16q23 | Unknown | 0,0045845800 | 1,1618066558 |
| *KCNJ13* | Potassium inwardly-rectifying channel, subfamily J, member 13 | 2q37 | Ion transport | 0,0046036700 | -1,0564393904 |
| *SLC7A6OS* | Solute carrier family 7, member 6 opposite strand | 16q22.1 | Unknown | 0,0046562500 | -1,0964499817 |
| *ELOVL4* | Elongation of very long chain fatty acids | 6q14 | Fatty acid biosynthetic process | 0,0047475600 | -1,0751664311 |
| *UQCRFS1* | Ubiquinol-cytochrome c reductase, Rieske iron-sulfur polypeptide 1 | 19q12-q13.1 | Electron transport | 0,0049343800 | -1,3964655659 |
| *NBN* | Nibrin | 8q21 | DNA damage checkpoint, cell proliferation | 0,0049490500 | 1,2928366482 |
| *BEX2* | Brain expressed X-linked 2 | Xq22 | Unknown | 0,0049986300 | -1,1084548446 |
| *YPEL5* | Yippee-like 5 (Drosophila) | 2p23.1 | Unknown | 0,0051152800 | 1,2652302901 |
| *FAIM* | Fas apoptotic inhibitory molecule | 3q22.3 | Apoptosis | 0,0051474500 | -1,1050180993 |

**Table S1. (continued)**

| **Gene symbol** | **Gene name** | **Chromosomal location** | **Biological process** | **P-value** | **Fold change** |
| --- | --- | --- | --- | --- | --- |
| *STAT1* | Signal transducer and activator of transcription 1 | 2q32.2 | Regulation of progression through cell cycle, immune response | 0,0051854400 | 1,2705031920 |
| *IL8* | Interleukin 8 | 4q13-q21 | Angiogenesis, immune response | 0,0053848600 | -1,9240629489 |
| *PIH1D2* | PIH1 domain containing 2 | 11q23.1 | Unknown | 0,0054837200 | -1,0243153941 |
| *EDC3* | Enhancer of mRNA decapping 3 homolog (S. cerevisiae) | 15q24.1 | Unknown | 0,0056516600 | -1,1112704744 |
| *TNFAIP3* | Tumor necrosis factor, alpha-induced protein 3 | 6q23 | Ubiquitin cycle, apoptosis, immune response | 0,0056746200 | 1,2149104686 |
| *FSCN1* | Fascin homolog 1, actin-bundling protein | 7p22 | Cell proliferation | 0,0058707000 | -1,1104465868 |
| *MGLL* | Monoglyceride lipase | 3q21.3 | Lipid metabolic process | 0,0060159500 | 1,4525219849 |
| *LOC652968* |  | 22q12 | Unknown | 0,0060943200 | -1,0947185474 |
| *GCNT2* | Glucosaminyl (N-acetyl) transferase 2, I-branching enzyme | 6p24.2 | Glycosaminoglycan biosynthetic process | 0,0061059700 | 1,2181569287 |
| *EGF* | Epidermal growth factor | 4q25 | Activation of MAPKK activity, immune response | 0,0061784600 | 1,4292516938 |
| *GLT25D2* | Glycosyltransferase 25 domain containing 2 | 1q25 | Lipopolysaccharide biosynthetic process | 0,0062007900 | -1,1030743229 |
| *HOP* | Homeodomain-only protein | 4q11-q12 | Regulation of transcription | 0,0063506800 | -1,1691013474 |
| *NT5C3* | 5'-Nucleotidase, cytosolic III | 7p14.3 | Pyrimidine nucleoside metabolic process | 0,0063729600 | 1,2438047226 |
| *RNF11* | Ring finger protein 11 | 1pter-p22.1 | Protein ubiquitination | 0,0065140800 | 1,2359239733 |
| *SLK* | STE20-like kinase (yeast) | 10q25.1 | Nucleotide-excision repair | 0,0065531400 | 1,1810990844 |
| *TAP2* | Transporter 2, ATP-binding cassette, sub-family B (MDR/TAP) | 6p21.3 | Protein complex assembly, immune response, antigen presentation | 0,0066674300 | 1,2265703652 |
| *GBP1* | Guanylate binding protein 1, interferon-inducible | 1p22.2 | Immune response | 0,0066915300 | 1,7005365329 |
| *GBP5* | Guanylate binding protein 5 (GBP5), mRNA | 1p22.2 | Immune response | 0,0066993200 | 1,4979609344 |

**Table S1. (continued)**

| **Gene symbol** | **Gene name** | **Chromosomal location** | **Biological process** | **P-value** | **Fold change** |
| --- | --- | --- | --- | --- | --- |
| *XRN1* | 5'-3' Exoribonuclease 1 | 3q23 | Cell cycle | 0,0068613700 | 1,2345626069 |
| *PTGDS* | Prostaglandin D2 synthase 21kDa (brain) | 9q34.2-q34.3 | Prostaglandin biosynthetic process | 0,0068699200 | -1,3189501147 |
| *TAS2R50* | Taste receptor, type 2, member 50 | 12p13.2 | Signal transduction | 0,0069844400 | 1,1668183661 |
| *HSPC159* | Galectin-related protein | 2p14 | Unknown | 0,0070339700 | 1,3686845716 |
| *ARL6* | ADP-ribosylation factor-like 6 | 3q11.2 | Small GTPase mediated signal transduction | 0,0071287800 | -1,0725091827 |
| *PDE4B* | Phosphodiesterase 4B, cAMP-specific (phosphodiesterase E4B) | 1p31 | Signal transduction | 0,0071689400 | 1,1907165330 |
| *OR2L3* | Olfactory receptor, family 2, subfamily L, member 3 | 1q44 | Signal transduction | 0,0074911100 | -1,2422882818 |
| *NR4A2* | Nuclear receptor subfamily 4, group A, member 2 | 2q22-q2 | Transcription | 0,0075340600 | 1,6270967063 |
| *KIAA1274* | KIAA1274 | 10q22.1 | Unknown | 0,0075434300 | -1,3458802019 |
| *OGG1* | 8-Oxoguanine DNA glycosylase | 3p26.2 | Base-excision repair | 0,0076458800 | -1,0936339995 |
| *CD97* | CD97 molecule | 19p13 | Cell motility, immune response, inflammatory response | 0,0079170700 | 1,1335122637 |
| *FRMD3* | FERM domain containing 3 | 9q21.32 | Cytoskeletal protein binding | 0,0081400100 | 1,2160562806 |
| *LRRC44* | Leucine rich repeat containing 44 | 1p31.1 | Unknown | 0,0082513600 | -1,0322768899 |
| *RAD23A* | RAD23 homolog A (S. cerevisiae) | 19p13.2 | Nucleotide-excision repair | 0,0085123400 | -1,1687043387 |
| *APP* | Amyloid beta (A4) precursor protein | 21q21.3 | Cellular copper ion homeostasis | 0,0085313800 | 1,1986579260 |
| *PXT1* | Peroxisomal, testis specific 1 | 6p21.31 | Unknown | 0,0085423800 | -1,0639423646 |
| *MPP7* | Membrane protein, palmitoylated 7 | 10p11.23 | Unknown | 0,0085852900 | 1,2653618455 |
| *NEXN* | Nexilin (F actin binding protein) | 1p31.1 | Regulation of cell migration | 0,0086569800 | 1,2841822829 |
| *GMPR* | Guanosine monophosphate reductase | 6p23 | Metabolic process | 0,0087634000 | 1,3104842366 |
| *UVRAG* | UV radiation resistance associated gene | 11q13.5 | DNA repair | 0,0088686000 | 1,1618710819 |
| *ADAMTS1* | ADAM metallopeptidase with thrombospondin type 1 motif, 1 | 21q21.2 | Proteolysis, regulation of cell proliferation | 0,0089007800 | -1,1392939296 |

**Table S1. (continued)**

| **Gene symbol** | **Gene name** | **Chromosomal location** | **Biological process** | **P-value** | **Fold change** |
| --- | --- | --- | --- | --- | --- |
| *ATP6V0A2* | ATPase, H+ transporting, lysosomal V0 subunit a2 | 12q24.31 | Ion transport, immune response | 0,0089238500 | -1,3372600073 |
| *CATSPER3* | Cation channel, sperm associated 3 | 5q31.1 | Ion transport, cell differentiation | 0,0090418000 | -1,1070573796 |
| *C5* | Complement component 5 | 9q33-q34 | Activation of MAPK activity, inflammation | 0,0093074200 | 1,1028679021 |
| *MAP4K2* | Mitogen-activated protein kinase kinase kinase kinase 2 | 11q13 | Protein amino acid phosphorylation, immune response | 0,0093747500 | 1,1135914195 |
| *GCH1* | GTP cyclohydrolase 1 (dopa-responsive dystonia) | 14q22.1-q22.2 | L-phenylalanine catabolic process, immune response | 0,0093934700 | 1,2451590187 |
| *ATP6V0E2* | ATPase, H+ transporting V0 subunit e2 | 7q36.1 | Ion transport | 0,0094295700 | -1,1157083879 |
| *AK123815* |  | 13q11 | Unknown | 0,0094493000 | -1,1195585660 |
| *FBXO10* | F-box protein 10 | 9p13.2 | Protein ubiquitination | 0,0094812400 | -1,2291406397 |
| *ZNF425* | Zinc finger protein 425 | 7q36.1 | Transcription | 0,0094925000 | -1,0742427199 |
| *HSCB* | HscB iron-sulfur cluster co-chaperone homolog (E. coli) | 22q12.1 | Protein folding | 0,0096215000 | -1,0823221672 |
| *GTF2F2* | General transcription factor IIF, polypeptide 2 | 13q14 | Transcription | 0,0096299800 | 1,2006785839 |
| *PGK1* | Phosphoglycerate kinase 1 | Xq13 | Glycolysis | 0,0097520800 | -1,1432968190 |
| *STAT2* | Signal transducer and activator of transcription 2 | 12q13.2 | Transcription, response to cytokines, immune response | 0,0098147500 | 1,3457309473 |
| *PCSK6* | Proprotein convertase subtilisin/kexin type 6 | 15q26.3 | Proteolysis | 0,0098520000 | 1,3444349942 |
| *C9orf91* | Chromosome 9 open reading frame 91 | 9q32 | Unknown | 0,0098952400 | 1,2555243422 |
| *PPCDC* | Phosphopantothenoylcysteine decarboxylase | 15q24.2 | Coenzyme A biosynthetic process | 0,0099829100 | 1,1998715757 |
| *GSX1* | GS homeobox 1 | 13q12.2 | Regulation of transcription, DNA-dependent | 0,0099942800 | -1,1355332887 |

neg. = downgeruguleerd in responders vs non-responders, pos. = upgereguleerd in responders vs non-responders
